# Rationale and Methods of Evaluation for ACHO, A New Virtual Assistant to Improve Therapeutic Adherence in Rural Elderly Populations: A User-Driven Living Lab

**DOI:** 10.3390/ijerph18157904

**Published:** 2021-07-26

**Authors:** Jeronimo Luengo-Polo, David Conde-Caballero, Borja Rivero-Jiménez, Inmaculada Ballesteros-Yáñez, Carlos A. Castillo-Sarmiento, Lorenzo Mariano-Juárez

**Affiliations:** 1 Department of Nursing, Faculty of Nursing & Occupational Therapy, University of Extremadura, 10003 Cáceres, Spain; jeronimolp@gmail.com (J.L.-P.); dcondecab@unex.es (D.C.-C.); lorenmariano@unex.es (L.M.-J.); 2 Department of Computer Systems and Telematics, Polytechnic School, University of Extremadura, 10003 Cáceres, Spain; brivero@unex.es; 3 Department of Inorganic, Organic Chemistry and Biochemistry, School of Medicine, University of Castilla-La Mancha, 13071 Ciudad Real, Spain; Inmaculada.BYanez@uclm.es; 4 Regional Center for Biomedical Research, University of Castilla-La Mancha, 02008 Albacete, Spain; 5 Department of Nursing, Physiotherapy and Occupational Therapy, School of Physiotherapy and Nursing, University of Castilla-La Mancha, 45071 Toledo, Spain

**Keywords:** active assisted living program, adherence, e-Health, living lab, voice assistant

## Abstract

Low therapeutic adherence is a concern for health professionals as it decreases therapeutic efficiency while increasing costs, especially in elderly populations. To increase therapeutic adherence in elderly populations, the technology applied in the medical devices that are used must be adapted to improve usability. This paper outlines the rationale behind, and methods applied to assess the usability of, ACHO (Assistant on Care and Health Offline), a voice assistant that provides elderly patients with reminders of medical appointments to attend and when they need to take their medication. This work is a descriptive, cross-sectional, observational study, and will include a three-phase (analysis, testing and refinement) multidimensional usability analysis of an initial prototype, in the setting of a user-driven Living Lab, which enables the needs and characteristics of the end users to be identified and incorporated into the prototype with each iteration, in which a multidisciplinary team of researchers and users will participate as co-creators. This methodology will allow us to develop a better prototype, increasing usability and, thus, increasing therapeutic adherence.

## 1. Introduction

Elderly patients are especially susceptible to the phenomenon of medication nonadherence, which can be defined as the extent to which recommendations for dosage and frequency of drug intake are met [1]. In fact, the WHO estimates that between 30% and 50% of patients do not follow treatments as prescribed [2]; although this is a complex parameter to measure, rates of medication nonadherence are higher in the elderly [3,4,5]. Aspects such as reminders of medical appointments and when medications should be taken are part of the concept referred to as therapeutic adherence, which is a very complex area.

In the past decade, technology has been increasingly used to improve therapeutic adherence and further support patients and medical–health services. Technological evolution in recent years has led to studies on electronic reminders using audio [6,7] and audiovisual devices [8,9] which, through warnings or alarms, unlock ever greater opportunities to apply technology in the field of adherence. Voice assistants can also be used to provide reminders which improve therapeutic adherence [10,11]. A voice assistant is a software agent that can perform tasks or services for an individual and that interacts through voice activation using an intelligent speaker device [12]. One of the first studies to investigate health-related information from voice assistants showed that Cortana (Microsoft), Google Now (Google), Siri (Apple) and S Voice (Samsung) responded inconsistently and incompletely to a variety of mental and physical health questions [13]. Subsequent studies have highlighted the high variability in the outcomes of implementing these assistants in the treatment of health problems, considering them to be a safety risk for patients and consumers [14,15].

Research points to the importance of adapting technology to the user experience by involving the end user in the development of the technology itself. Thus, by making these adaptations according to the needs of the elderly target group, considerable increases can be observed [16,17]. Furthermore, if these modifications improve the usability of the system, they are likely to increase adherence as well, since a direct relationship between the low usability of a device and lower adherence has previously been identified [18]. To overcome the challenges that can arise when working with technological devices and older people, a working methodology known as a “Living Lab” can be employed. The Living Lab concept dates from the 1990s, and refers to an approach to innovation which involves a group of researchers collaborating with target users as co-creators in the development and validation of new products. The Living Lab paradigm, thus, encourages innovation through an iterative process, usually divided into 3–6 stages, in which a product is gradually refined from a user-focused perspective [19].

This paper outlines the rationale and methods for the design of a usability assessment process for ACHO (Assistant on Care and Health Offline), a voice assistant developed to increase therapeutic adherence in elderly populations. The development process for ACHO employs a user-driven approach involving a multidisciplinary team and a three-stage optimization process governed by a variety of complementary controls. The design process requires the collaboration of end users in order to adapt the assistant’s features to the needs and characteristics of the target users, which is an important factor in improving the ultimate user experience and ensuring the highest standards of safety in application.

This work presents a study protocol for the usability evaluation of a voice assistant, ACHO. This voice assistant aims to improve therapeutic adherence in rural elderly populations by developing functions to remind patients to attend medical appointments and to take their medication. By employing a Living Lab methodology, the voice assistant is developed following principles set out by European programs such as the Active and Assisted Living Programme.

## 2. Materials and Methods

This study protocol presented the usability evaluation of a voice assistant by means of a mixed quantitative–qualitative method through a three-stage optimization process.

ACHO is a voice assistant designed to improve the therapeutic adherence of older people in rural areas, focusing so far on aspects such as reminders of medical appointments and medication times. The voice assistant is based on two main components: a mobile application, which provides information on appointments and prescriptions, and a voice assistant, which is responsible for interacting directly with the elderly patient. The initial prototypes of both were developed by a multidisciplinary research group, including team members from the fields of nursing and anthropology, as well as computer engineering. In a previous publication, we already described how the ACHO prototype was developed and implemented through the tools of qualitative research work [20].

**Figure 1:**
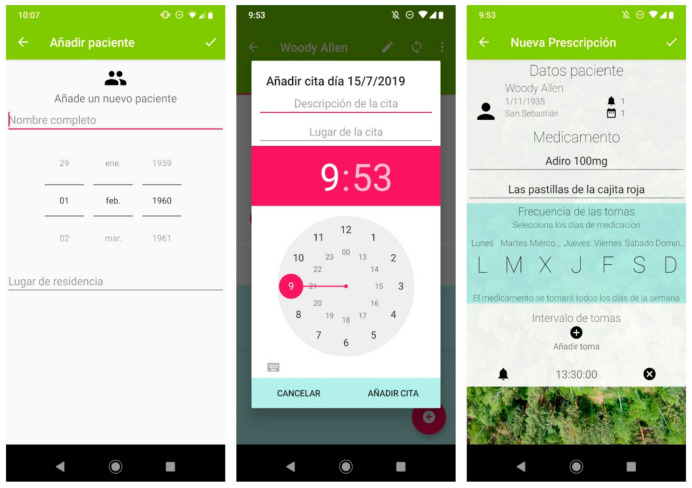
A selection of screenshots from the app. The application allows information on multiple patients to be stored (**left panel**). It includes a calendar (**central panel**) where the date, time, location and description of the next medical appointment can be specified. The pharmacological reminder function (**right panel**) allows the health professional to specify the name of the medicine, a brief personalized description of it, the days on which it is to be taken, and at which times. The application includes a synchronization button for the encrypted transmission of the data to the voice assistant via Bluetooth.

**Figure 2:**
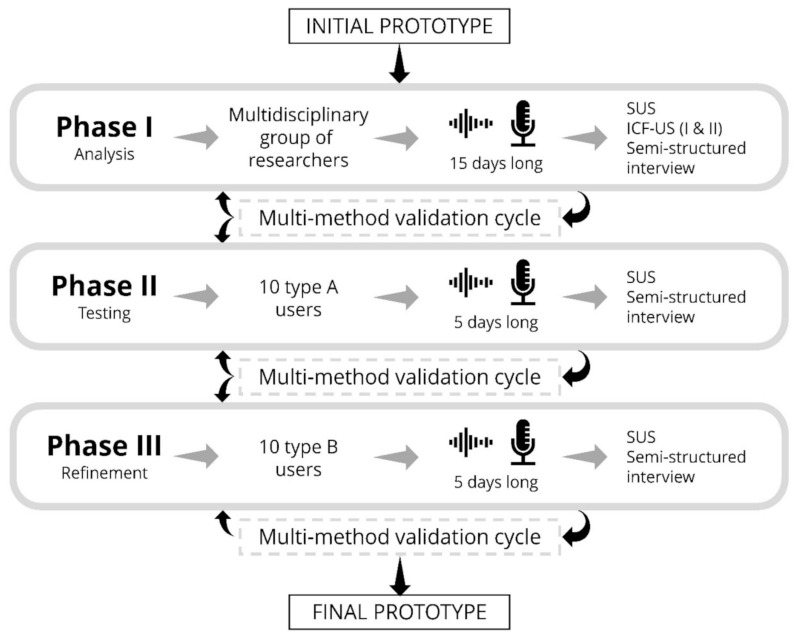
Scheme of the phases for usability evaluation of ACHO. The diagram shows the users involved and estimated duration for each phase, along with the tools that will be used to measure usability and evaluate user experience. The transition from one phase to the next will be controlled by multi-method optimization cycles.

The voice assistant was developed based on ‘Snips’ technology (Sonos, Paris, France) as this allowed the developers some freedom in the design, while maintaining data privacy. Another advantage was that it can be installed on a Raspberry Pi computer. These technical specifications do not directly influence the user’s interaction with the device. The system flow involves three main phases: (1) health professionals enter patient information in the application (Figure 1), (2) the data are transferred from the app to the voice assistant via Bluetooth, and (3) the voice assistant gives the user reminders of events.

The voice assistant reminds users when they must take their medication throughout the day, or if they have a medical appointment the next day. After the reminder to take the medication, ACHO will ask the user whether they have taken the medication. If there is no positive answer, the voice assistant saves this information so that a family member or health professional can check it later.

The current prototype includes some innovative features tailored to the rural elderly population, such as: (i) being mains-powered, it is always operational, as participants prefer technologies that can be used for several days or weeks without having to be recharged, as the need for recharging can disrupt the patient’s daily activities [21]; (ii) ACHO does not require an internet connection, so it is ideal for rural contexts; (iii) no smartphone is needed for daily operation, as some elderly people report a dislike of this technology (the aim is that the patient’s relatives, caregivers or even, in the future, healthcare professionals, will be in charge of updating the patient’s medication regimen via their smartphones) [22]; (iv) ACHO allows custom names to be used for each drug in the alerts, which reduces the occurrence of errors in taking medication and increases adherence [23].

### 2.1. Participants

For the purposes of evaluating the usability of the medication and medical appointment reminders of our initial prototype, we defined two types of volunteers—type A and type B users (five men and five women per type, from a total of twenty participants)—who will participate in phases 2 and 3, respectively, of the evaluation.

The type A group will consist of people over 65 years of age, coming from rural areas and with no cognitive or sensory impairment, selected on a non-random basis following a recommendation of suitability by health service professionals in the selected location. An essential criterion for inclusion of a member in the selected sample is that they must have the capacity to recognize and report on their own possible failures, “critical incidents” (those events in professional practice that caused us perplexity, created doubts, produced surprise or annoyed or disturbed us because of their lack of coherence or because they presented unexpected results) [24] and problems interacting with the prototype.

Type B users will be people over 65 years of age from rural areas, with the only inclusion criteria being that they are not taking any critical medication (medication that, if taken outside the prescribed schedule, would lead to serious health problems or a significant destabilization of the patient’s health). These will be selected randomly from a list of suitable users provided by health service professionals in the selected location.

### 2.2. User-Driven Living Lab Design

The proposed method incorporates an inductive qualitative analysis [25]. This approach was chosen in order to capture and analyze feedback in broad terms, and to reduce the potential for bias which may be introduced with more systematic approaches [26]. At the start of each phase, the participants will start the evaluation from scratch. Evaluation categories from previous users will then be incorporated and discussed, in order to aid the triangulation process.

The usability of the system will be evaluated using a combination of distinct but complementary methods that will facilitate a multidimensional evaluation of the prototype. The study will employ observational, descriptive and mixed approaches and, in accordance with the available evidence of previous studies that advocates a cyclical sequence of analysis, prototyping, testing and refinement of the mechanisms of interaction with the user [27,28] will include a usability analysis across several phases, with multiple data collection and monitoring points. In this regard, it is important to emphasize that the progression from one phase to another will be limiting, as the study will not advance to the next stage without having satisfied the control points established in the current phase.

The three phases that we decided to include in the design of the optimization process are described below.

#### 2.2.1. Phase I: Analysis

The first phase of the evaluation will aim to determine whether our product is sustainable in terms of its interface and functionality. To this end, a total of 10 researchers, representing various areas of expertise, from the International Institute for Research and Innovation on Ageing, will evaluate the usability of the device over a period of 15 days. The researchers will be given a series of script-based tasks that they will have to perform at least three times a day, simulating the normal pattern of medication intake.

#### 2.2.2. Phase II: Testing

In the second phase of the prototype evaluation, we intend to collect information on usability and user satisfaction for the group of “type A” users defined above. This will involve a physical implementation of the prototype in real but controlled contexts. In this phase, which will last 5 days, the characteristics of the medications taken by the type A users (none of the medications will be critical) will be previously loaded onto the device by health professionals with the assistance of the researchers.

The users will be fully trained beforehand in the actions to be carried out, and will be accompanied by “Observation Units” in at least one of the three scheduled daily interactions with the device. These Observation Units will be composed of researchers who will observe and evaluate the process of use and interaction in the user context, which will allow us to collect important information to help understand which changes must be performed in the environmental factors to better adapt the prototype to its users and improve its functionality [29]. In addition, the Observation Units will be responsible for recording so-called critical incidents, i.e., any situation that deviates from normality [30].

#### 2.2.3. Phase III: Refinement

The third and final phase of the evaluation of the pilot test aims to assess usability under normal, uncontrolled operating conditions. This phase will be very similar in character to the previous one, except for the type of users; in this phase, type B users, with prior training in the actions to be carried out and the objectives to be pursued, will be accompanied by Observation Units in the scheduled daily interactions.

### 2.3. Outcome Measures and Data Collection

Here, we will present the outcome measures and data collection for each of the phases described in the previous section. Our methodology to evaluate prototype usability will employ two different scales: the System Usability Scale (SUS) and the International Classification of Functioning, Disability and Health Usability Scale (ICF-US).

#### 2.3.1. System Usability Scale

This scale was developed in 1986 by the Digital Equipment Corporation as part of the application of usability engineering to public domain and open source office systems [31]. In addition, it is a very simple tool to use, and can be adapted for use in different situations. Despite the extraordinary simplicity of the scale, it has demonstrated highly robust and solid results, which is why it is one of the most widely used methods of measuring usability [32].

#### 2.3.2. International Classification of Functioning, Disability and Health Usability Scale

Our evaluation of the usability of the prototype will also apply the ICF-US I scale and the ICF-US II subscale [30,33], which have the advantage of being based on concepts and terminology established by the World Health Organization, meaning that they are universally accepted [34]. The ICF-US I scale allows the identification of general usability problems, while the ICF-US II subscale aids the identification of possible barriers and/or enablers, as well as more precisely identifying those elements that may require further work to improve the device. The advantage of this scale is that once a barrier is recognized, it is possible to identify the characteristic that gives rise to it, in order to determine what can be improved. On the other hand, characteristics identified as facilitators can be highlighted as good practice for future development [35]. The complementary use of these two instruments will allow us to make a more detailed assessment of the usability of our prototype.

#### 2.3.3. Semi-Structured Interviews

The anthropologists participating in this research project will be responsible for conducting an individual, ad hoc semi-structured interview (Table 1) with each user of the prototype. The objective of these interviews will be to provide deeper qualitative insight into the users’ experiences and interaction with the prototype (number of reminders, tone of voice, naming of the drugs, acceptance of the system, message saturation, errors, hearing problems and other subjective elements).

#### 2.3.4. Multi-Method Optimization Cycles

For each phase of this development study, quality parameters were established that must be satisfied as a necessary and sufficient condition to advance to the next phase of the process (Figure 2). For each of the phases, a final score greater than 68 on the SUS scale, averaged over all users, must be achieved. This is the threshold established in the scientific literature to demonstrate the correct usability of a product [36]. In addition, all semi-structured interviews must be completed, and any improvements suggested from the analysis of the evaluation instruments should be incorporated into the device [37,38]. After completing this process and incorporating the necessary improvements, the researchers should assess the need for a new evaluation cycle according to the extent of the changes performed.

There is an extra requirement that to pass Phase I, as it uses the ICF-US scale, the average score awarded by researchers participating in the evaluation of the device must be higher than 10 on the ICF-US I scale, this being the threshold established in the scientific literature to demonstrate the correct usability of a product [39]. This phase of the evaluation process also specifies that the results obtained using the ICF-US II instrument must be analyzed.

Once the optimization cycle proposed will be completed, the possibility of incorporating new functionalities will be assessed, in which case the optimization process would be the same as the one described here. Otherwise, the next phase would involve assessing the safety of the use of the prototype with respect to the medical appointments and medication reminders provided.

### 2.4. Data Analysis

Data will be collected anonymously, and participants will receive all the information generated by the study. Quantitative data will be stored and analyzed in SPSS (SPSS Inc., Chicago, IL, USA), version 22. Qualitative data will be stored and categorized in the Dedalo Software Platform for Oral History and Intangible Cultural Heritage Management.

Qualitative data will be handled and analyzed following previously reported protocols [40]. Briefly, semi-structured interviews will be conducted by a team of three researchers. The audio of all interviews will be recorded, and field notes will be taken during the interviews. Transcripts of the audio recordings will be conducted, supported by the field notes, preserving the anonymity of the participants. The transcribed interviews will be analyzed with the qualitative data analysis software ATLAS.ti (Scientific Software Development GmbH, Berlin, Germany, version 7.5.7 for Windows). The empirical material will be analyzed using the constant comparison method, inductive analysis, and triangulation. The analysis shall meet all 31 of the criteria defined in the COREQ (Consolidated Criteria for Reporting Qualitative Research) checklist [41].

## 3. Discussion

The scientific literature has described a number of methodologies and tools to ensure the quality of usability of a service [27,42,43]. Evaluating the usability and user experience of a piece of technology is an essential step for it to have any significant degree of acceptance and meet its objectives [42]. This is even more important when considering older people because of the particular characteristics of this age group, which were identified as a major factor in the adoption of health-related technologies [44]. Many previous products similar to ours have not been widely accepted because they do not take these types of issues into account and are designed from a predominantly technical point of view [45]. It is, therefore, especially important to seek empirical evidence on how to improve the usability of different devices [46].

For this reason, it was decided to carry out this evaluation within the framework of a Living Lab, which will allow us to understand the needs and preferences of and technological challenges faced by end users by involving them in the development of the prototype [47], thus, facilitating its use in the long term [48]. Living Labs emphasize the iterative process of experimentation and learning by increasing the understanding of real-life problems [49]. The resulting design changes can, thus, reduce errors and enhance usability and user acceptance, so it is the technology that adapts to the user and not the user who has to adapt to the technology [38,50]. In our opinion, this method allows for a more realistic evaluation of the environmental and holistic factors affecting the user. As indicated by Bevan et al., the implementation of user-centered methods ensures that ‘real products can be used by real people to perform their tasks in the real world’ [51].

The semi-structured interview is a key research tool in the field of anthropology. Nevertheless, there is no literature consensus on the optimal degree of openness of the questions which comprise the semi-structured interview. While some authors argue that the interview should not follow any type of scheme [52]; others recommend including a list of themes and sub-themes [53]; yet others advocate the development of a script of topics, standard questions and thematic progression of interviews [54]. The present design advocates an intermediate position, maintaining an order for the set of thematic blocks while maintaining the openness of the individual questions depending on the context, with a focus on obtaining candid answers from the participant, supplemented by a final section inviting stories of concrete experiences. By conducting a qualitative evaluation of the device, the research tools and the sampling of participants must be in line with qualitative research methods. Thus, we believe that the final sample of 20 participants is the ideal sample size that will allow us to analyze the usability and acceptance of the product. As Sandelowski’s classic text points out [55]: “Determining adequate sample size in qualitative research is ultimately a matter of judgment and experience in evaluating the quality of the information collected against the uses to which it will be put”.

The usability study described above illustrates the importance of training multidisciplinary teams in the development of technological applications in clinical practice, in order to understand not only the technical aspects of a device, but also user perspectives. In this regard, the inclusion of end users in the development of the prototypes results in a more useful prototype that takes into account the needs and characteristics of the target population.

### Limitations

The information stored in ACHO is updated via Bluetooth by means of an app. Therefore, the information must be entered into the app by an administrator. This means that ACHO is affected by similar limitations to any other tool that requires the digitalization of health systems; these primarily relate to confidentiality, data security, communication between systems, maintenance, and training of health personnel. Another limitation of our study may be the lack of use of technology by the elderly. Precisely for this reason, we tried to design a technological device that depends as little as possible on the use of smartphones by the end users, since older adults, especially in our area, report a very limited use of technology. Likewise, this evaluation also aims to find out to what extent this technological device is or is not accepted by the end users. In addition, we are aware of the limitations of following a mixed approach and working in an interdisciplinary setting. Although it does have obvious advantages, interdisciplinary work poses a challenge in terms of integrating diverse assessment tools and strategies that practitioners of individual disciplines are not always familiar with.

## 4. Conclusions

At the core of our design process is a multimethodological approach to evaluate the usability and user experience of a voice assistant by involving target users as co-creators in a Living Lab. This design process will allow us to increase usability as well as user satisfaction by including improvements that target users consider relevant, and by doing so, to create a product in which technology is adapted to the user.

In terms of future research directions, in the short term we are planning to add different voices to the assistant (e.g., relatives, doctors, nurses) to enhance the user experience, and in the medium term to customize the information provided by the assistant according to the context by collecting epidemiological data as well as other public health alerts relevant to the target user.

## Figures and Tables

**Table 1:** Questions used as a reference to guide the ad hoc semi-structured interviews.

Thematic Areas/Theoretical Questions	Sample Questions (Flexible Examples)
General evaluation. Level of satisfaction with usability	Tell me what you thought of ACHO. Tell me about these days with ACHO. What did you like most and least?
Learning experiences	What has it been like starting a relationship with ACHO? Tell me about the first days
Achieving the expected results	Could you tell me if it has helped you? Can you give me examples? Did you expect it to be like this or different in any way?
Assessment of interaction/communication	What has it been like talking to ACHO? How did you start? What would you say about this kind of communication?
About the voice	What do you think of the voice? Would you prefer a different one?
About reminders	What did you think of the number of reminders? Too few, too many?
About the context	Where did you place ACHO? Why?
Problems	Could you tell me about anything that did not work as intended?
Improvements	What would you ask ACHO to do that it does not do now?
